# Urinary levels of Hepatocarcinoma-intestine-pancreas/Pancreatitis-associated protein as a diagnostic biomarker in patients with bladder cancer

**DOI:** 10.1186/1471-2490-12-24

**Published:** 2012-09-04

**Authors:** Yujiro Nitta, Hiroyuki Konishi, Tetsuya Makino, Tomoaki Tanaka, Hidenori Kawashima, Juan L Iovanna, Tatsuya Nakatani, Hiroshi Kiyama

**Affiliations:** 1 Department of Urology, Graduate School of Medicine, Osaka City University, Osaka, Japan; 2 Department of Functional Anatomy and Neuroscience, Graduate School of Medicine, Nagoya University, Nagoya, Japan; 3 INSERM U.624, Stress Cellulaire, France

**Keywords:** Bladder cancer, Urinary marker, HIP/PAP, ELISA, ROC

## Abstract

**Background:**

To assess the possibility of hepatocarcinoma-intestine-pancreas/pancreatitis-associated protein (HIP/PAP) as a biological marker for detecting Bladder cancer (BCa), we examined the expression of HIP/PAP in both BCa specimens and BCa cell lines and measured HIP/PAP levels in urine from patients with BCa.

**Methods:**

HIP/PAP expression in BCa samples was evaluated by western blot analysis, and urinary levels of HIP/PAP in patients with BCa were measured by enzyme-linked immunosorbent assay. Urine samples were collected from 10 healthy volunteers and 109 with benign urological disorders as controls, and from 101 patients who were diagnosed with BCa.

**Results:**

HIP/PAP was highly expressed in BCa samples as compared with control bladder. Urinary HIP/PAP concentrations were significantly higher in BCa patients than in controls (median value; 3.184 pg/mL vs. 55.200 pg/mL, P <0.0001, by Mann–Whitney *U* test). Urinary HIP/PAP levels in BCa patients correlated positively with pathological T stages and progression-risk groups among non-muscle invasive BCa (P = 0.0008, by Kruskal-Wallis test). Regarding the recurrence-risk classifications of non-muscle invasive BCa, the urinary levels of HIP/PAP were significantly higher in the intermediate than in the low risk group (P = 0.0002, by Mann–Whitney *U* test). Based on a cut-off of 8.5 pg/mL, the ability of urinary HIP/PAP levels to detect BCa had a sensitivity of 80.2%, specificity of 78.2%, positive predictive value (PPV) of 75.7%, and negative predictive value (NPV) of 82.3%.

**Conclusions:**

HIP/PAP was abundantly expressed in BCa, and the urinary levels of HIP/PAP could be a novel and potent biomarker for detection of BCa, and also for predicting the risks of recurrence- and progression-risk of non-muscle invasive BCa. A large scale study will be needed to establish the usefulness of this biomarker.

## Background

Urothelial carcinoma is the most common Bladder cancer (BCa). In 2008, an estimated 38,6300 cases were newly diagnosed with bladder cancer and there were 150,200 deaths due to bladder cancer worldwide. [1] Early detection of primary and recurrent bladder cancers may greatly reduce the mortality rate. The cystoscopic examination is a feasible procedure for detection of BCa. However, the examination is invasive for patients. On the other hand, urine cytology is the most widespread method for identifying BCa noninvasively despite its low sensitivity for detecting low grade BCa. [2] In recent years, numerous urine-based bladder tumor markers, including bladder tumor antigen (BTA) and nuclear matrix protein 22 (NMP22), have been reported as powerful tools for the detection of BCa. In particular, NMP22 has been widely used as a urine marker for BCa of good sensitivity in comparison with urine cytology. Numerous studies have demonstrated that NMP22 has a superior sensitivity (46–88% and 40–85%, respectively) to urine cytology. [3] However, there remain differences among reports yet, and the sensitivity is not fully satisfactory.

A protein called regenerating gene/regenerating islet-derived (Reg) was originally identified in regenerating pancreatic islet cells. [4] To date, several Reg family proteins have been identified and described under various nomenclatures such as pancreatitis-associated protein (PAP) and hepatocarcinoma-intestine-pancreas (HIP). They are broadly categorized into four groups, namely, type I to IV. [5,6] In humans, six gene products, Reg-Iα, Reg-Iβ, Reg-III (corresponding to Reg-IIIα in the mouse), HIP/PAP (corresponding to Reg-IIIβ in the mouse), Reg-IIIγ and Reg-IV have been identified. [7,8] These members have been shown to be secretory proteins that play a key role in both tissue regeneration and inflammation in digestive organs. [9-11] Some of them are also highly expressed in some cancers and function as proliferative, tropic or anti-apoptotic factors in cancer cells. [10,12] From the aspect of their characteristics as secretory proteins, the examination of Reg family proteins in body fluids (e.g., serum, urine) may contribute to the detection of various cancers. [13-17] In fact Reg-IV was demonstrated to be a potent screening marker for gastric and pancreatic cancer. [18,19] Furthermore, proteomic screening revealed urinary levels of Reg-I to be a potential marker for BCa [20].

We previously reported that the interstitial cystitis (IC) induced expression of some Reg family members in Cyclophosphamide-induced cystitis model of rat, and demonstrated that PAP-III (Reg-IIIγ in humans) was expressed in the urothelium of the bladder and, on the other hand, that PAP-I (HIP/PAP in humans) was expressed in primary afferent neurons of dorsal root ganglia that innervate the bladder. [21] Recently, we also reported urinary levels of HIP/PAP to be significantly higher in painful bladder syndrome (PBS)/IC patients than in healthy controls. [22] Furthermore, protein expression of HIP/PAP was very high in the bladder urothelium of PBS/IC patients. [22] In this study, based on these findings, we investigated the possibility of high levels of HIP/PAP in urine from BCa patients, and revealed a possibility of urinary HIP/PAP as a diagnostic biomarker of BCa. We also evaluated the diagnostic accuracy of urinary HIP/PAP as compared with conventional markers, such as urinary NMP22 and BTA.

## Methods

### Patients and samples

We followed our institution’s ethics guidelines and the study was approved by the ethics committee of Osaka City University Hospital. All patients provided informed consent.

Urine samples were collected from 10 healthy volunteers and 109 with benign urological disorders as controls, and from 101 patients who were diagnosed with BCa at Osaka City University Hospital between 2008 and 2011 (Table1). Voided urine samples were collected into a sterile container at the urological section. Both urinary NMP-22 and BTA levels were measured and the cytology was examined using freshly voided urine immediately after the samples were obtained. The rests kept at 4°C. They were transported to our laboratory and centrifuged at 800 × g for 10 minutes. The supernatants were stored at -80°C until the following analyses were performed.

**Table 1:** Patient characteristics

**BCa**	
Total number	101
Median age (range) (years)	70.3(49-88)
Sex	
Male	75
Female	26
T-category	
Ta	48
T1	21
T2	16
≧T3	12
Grade	
G1	8
G2	54
G3	25
**Controls**	
Total number	119
Median age (range) (years)	60.2(29-84)
Sex	
Male	86
Female	33
Healthy volunteers	10
Benign urological disease	109
BPH	41
LOH syndrome	29
NGB	18
Urolithiasis	8
Severe cystisis	6
Renal cysts	5
Nutcracker syndrome	2

Total numbers of the patients do not always add up to 101 because clinical information was incomplete for some individuals.

Tumor tissues were collected after transurethral resection, and cystectomy. As a control tissue, normal bladder was collected from patients who had undergone cystectomy for localized BCa. The collected specimens were immediately frozen and stored at -80°C in a freezer.

### Cell lines and cell culture

Human bladder cancer cell lines T24, TCCSUP, RT4, HT1376 and UMUC-3 were obtained from the American Type Culture Collection (Manassas, VA, USA). All cell lines were maintained in RPMI 1640 (Sigma, St. Louis, MO, USA) supplemented with 10% fetal bovine serum, 100 U/mL of penicillin and 100 μg/mL of streptomycin (Gibco, New York, USA) at 37°C in a humidified atmosphere containing 5% CO_2_.

### Western blotting

Tumor and normal tissue samples were homogenized in lysis buffer containing 40 mM Tris base, 8 M urea and 2% CHAPS, and centrifuged at 10,000× g for 20 min at 4°C. The supernatants were aliquoted and stored at -80°C after measurement of protein concentrations. Cells were harvested and whole-cell lysate were prepared using the PRO-PREP protein extraction solution (iNtRON Biotechnology, Gyeonggi-do, Korea) in accordance with the manufacturer’s instructions. Protein samples (100 μg of each protein) were treated at 55°C for 10 min in 2% SDS-treating solution containing 5% β-mercaptoethanol and separated in 10% SDS-polyacrylamide gels and transferred onto nitrocellulose membranes. Membranes were blocked for 1 h at room temperature with Tris-buffered saline (TBS) containing 0.05% Tween 20 and 1% BSA, and incubated overnight at 4°C with the following primary antibodies; anti-HIP/PAP (1:400 dilution) [23] and anti-β-actin (1:8000 dilution, Abcam, Cambridge, UK). Immunoblots were washed with TBS containing 0.05% Tween 20 and incubated with secondary antibodies conjugated with horseradish peroxidase anti-mouse IgG or anti-rabbit IgG (Santa Cruz Biotechnology, Santa Cruz, CA, USA) for 1 h at room temperature. Immunoreactive proteins were visualized using the ECL detection system (Pierce, Rockford, IL,USA).

### Enzyme-linked immunosorbent assay (ELISA) for urinary HIP/PAP

Urinary HIP/PAP concentrations were measured by ELISA (Dynabio, Marseille, France). Urine samples were pipette into wells precoated with anti-HIP/PAP antibody according to the manufacturer’s instructions. After incubation for 3 hours at room temperature, the plates were rinsed with washing buffer. They were then incubated with biotinylated anti-HIP/PAP antibody. The plates were rinsed, incubated with avidin-peroxidase, and developed with tetramethylbenzidine substrate. Development was stopped with sulfuric acid, and absorbance at 450 nm was determined with an automatic plate reader. To examine the cross-reactivates of this kit, Reg-Iα (Abcam Ltd., Cambridge, UK), Reg-Iβ (Abcam Ltd.) and Reg-IV (R&D Systems, MN, USA) were measured.

### Measurement of urinary NMP-22 and BTA

Both urinary NMP 22 and BTA levels are measured in freshly voided urine samples. Measurement of urinary NMP-22 levels was requested of SRL, Inc. (Tokyo, Japan) and followed by measurement with the UNMP-22 test kit (Inverness Medical Japan, Tokyo, Japan). The cut-off value was 12.0 U/mL. Evaluation of urinary BTA levels was also requested of SRL, Inc. and followed by measurement with the BTA kit (TFB Inc,Tokyo, Japan). The BTA test is a latex agglutination assay for qualitative detection in urine.

### Statistical analysis

Mann–Whitney *U* test nonparametric analysis was performed for comparison of urinary HIP/PAP concentrations between two groups. The Kruskal-Wallis test was performed for comparison of urinary HIP/PAP concentrations among three or more different groups. P-values less than 0.05 were considered statistically significant. Receiver operating curve (ROC) analyses were used to define the optimal diagnostic cut-off as well as the diagnostic performance given by the area under the curve (AUC). JMP software (version 9.0.0; SAS Institute Inc, Cary, NC) was used for statistical analyses.

## Results

### HIP/PAP expression in the BCa specimens and BCa cell lines

**Figure 1:**
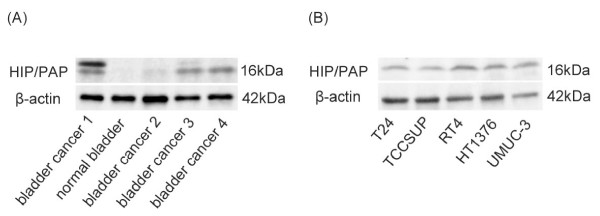
**Expression of HIP/PAP protein in tissue samples and BCa cells.** (**A**) Expression of HIP/PAP protein in a normal bladder tissue and BCa specimens. (**B**) Expression of HIP/PAP protein in human BCa cell lines. Protein samples were prepared from each specimen and cell line and then analyzed by western blotting using anti-HIP/PAP antibody to detect HIP/PAP protein (upper panel). To demonstrate equal loading amounts of samples, western blotting using anti-β-actin antibody was also performed (lower panel).

As shown in Figure1A, western blot analysis using the anti-HIP/PAP antibody revealed that the expression of HIP/PAP protein was detectable in BCa samples. On the other hand, HIP/PAP protein expression was not detected in a normal bladder tissue (Figure1A). Furthermore, HIP/PAP was highly expressed in five BCa cell lines derived from low grade (RT4), high grade (HT1376), invasive (T24, UM-UC-3) and metastatic (TCCSUP) tumors (Figure1B).

### Urinary HIP/PAP levels in relation to the pathological grade of BCa

**Figure 2:**
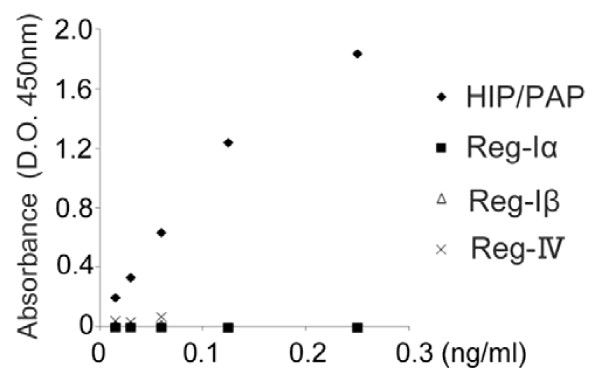
**Specificity of the HIP/PAP ELISA system.** The ELISA system had no or almost negligible cross-reactivity with Reg-Iα, -Iβ, and -IV.

**Figure 3:**
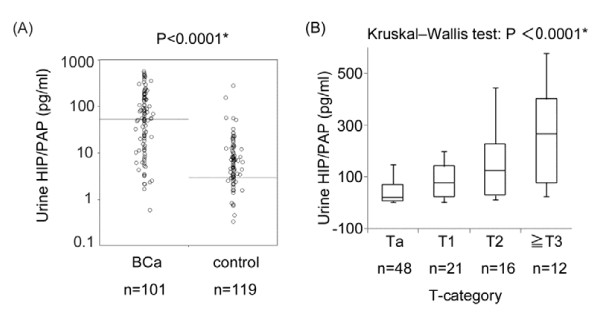
**Urinary levels of HIP/PAP determined by the ELISA system.** (**A**) Urinary HIP/PAP levels in BCa patients and controls. Bars represent median levels. The median urinary HIP/PAP concentration in BCa patients (median: 55.20 pg/mL, 25th and 75th percentiles: 11.91 and 150.96) was significantly higher than that in controls (median: 3.18 pg/mL, 25th and 75th percentiles: 0.00 and 8.28) (Mann–Whitney *U* test, P <0.0001). Asterisk indicates statistically significant P-values (P <0.05). (**B**) Correlation of urinary HIP/PAP levels in BCa patients with pathological stage. Patients were divided into four groups (Ta,T1,T2, ≥T3). The levels of urine HIP/PAP correlated proportionally with progression of T stage (Kruskal-Wallis test, P < 0.0001).

Urinary levels of HIP/PAP in both BCa patients and controls were measured using the HIP/PAP ELISA system. Because Reg family members have highly similar structures, we initially examined whether the ELISA system cross-reacted with other family members such as Reg-Iα, -Iβ, and -IV, whose expressions were previously reported in some malignant tissues. [13,16,18-20] The ELISA system detected only HIP/PAP and had no or almost negligible cross-reactivity with other human family members (Figure2). As shown in Figure3A, the urinary HIP/PAP concentration in the BCa group was significantly higher than that in controls (median value; 3.184 pg/mL vs. 55.200 pg/mL, P < 0.0001). There was a significant positive correlation between urinary HIP/PAP levels in BCa patients and their pathological T stages (Ta, T1, T2, ≥ T3) (P < 0.0001) (Figure3B).

### Urinary HIP/PAP levels in relation to recurrence-risk and progression-risk classifications of non-muscle invasive BCa

**Figure 4:**
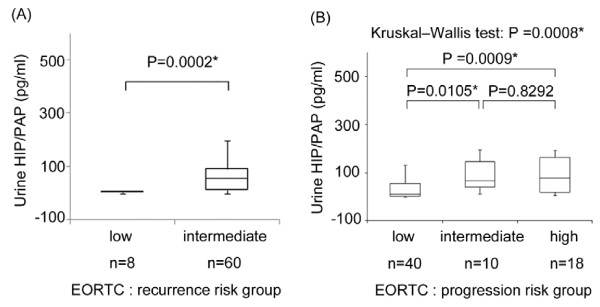
**Urinary HIP/PAP levels in relation to recurrence-risk and progression-risk classifications of non-muscle invasive BCa.** (**A**) Urinary levels were significantly higher in the intermediate risk group than in the low recurrence-risk group of non-muscle invasive BCa (Mann–Whitney *U* test, P = 0.0002). (**B**) Urinary HIP/PAP levels correlated significantly with increased risk of progression in non-muscle invasive BCa cases (Kruskal-Wallis test, P = 0.0008).

Recurrence-risk and progression-risk scores are calculated for each patient according to the European Organisation for Research and Treatment of Cancer (EORTC) definition. These factors comprise tumor grade, tumor stage, tumor size, numbers of tumor, earlier recurrence rate, and the presence of carcinoma *in situ*. Based on these scores, patients were considered to have low, intermediate, or high risk for recurrence and progression. With regards to the recurrence-risk classification of non-muscle invasive BCa, urinary levels of HIP/PAP in the low risk group were significantly lower than those in intermediate risk group (P = 0.0002) (Figure4A). In this study high recurrence-risk group did not exist. Moreover, the levels of urinary HIP/PAP showed a significant positive correlation with progression-risk groups among non-muscle invasive BCa cases (P = 0.0008) (Figure4B). In particular, urinary levels of HIP/PAP in the low progression-risk group were significantly lower than those in the intermediate and high progression-risk group (P = 0.0105 and P = 0.0009, respectively) (Figure4B).

### Comparison of sensitivity, specificity, positive predictive value (PPV) and negative predictive value (NPV) of urinary HIP/PAP with those of urinary NMP-22 and BTA regarding the prediction of BCa

**Figure 5:**
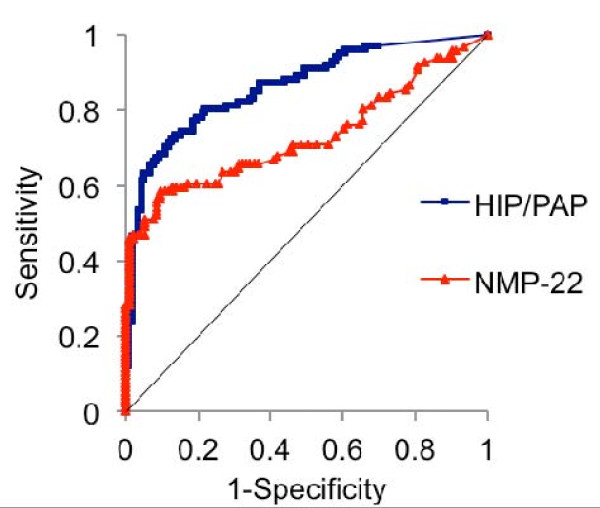
**Ability of urinary levels of HIP/PAP and NMP-22 to predict BCa using ROC analysis.** The AUC of HIP/PAP and NMP-22 were 0.863 (95% CI, 0.814-0.912) and 0.729 (95% CI, 0.655-0.803), respectively.

The possibility of urinary HIP/PAP levels predicting BCa was evaluated by ROC analysis (Figure5). As results of this calculation, a cut-off of 8.5 pg/mL was determined and the area under the ROC curve (AUC) was 0.863.

On the basis of this cut-off value, urinary HIP/PAP levels showed a sensitivity of 80.2%, specificity of 78.2%, PPV of 75.7%, and NPV of 82.3% (Table2). On the contrary, urinary levels of NMP-22 and BTA showed sensitivities of 52.1% and 34.7%, specificities of 93.5% and 96.1%, PPV of 89.3% and 91.7%, and NPV of 65.2% and 54.1%, respectively (Table2). Though urine cytology had a sensitivity of 38.8% (data not shown), we could not calculate specificity, PPV and NPV because few control patients underwent urine cytology.

**Table 2:** Sensitivity, specificity, PPV and NPV of urine markers HIP/PAP, NMP22 and BTA

	**HIP/PAP [95% CI]**	**NMP-22 [95% CI]**	**BTA [95% CI]**
Sensitivity (%)	80.2 [71.4 - 86.8]	52.1 [42.2 - 61.8]	34.7 [25.9 – 44.7]
Specificity (%)	78.2 [69.9 - 84.6]	93.5 [86.5 - 97.0]	96.1 [89.0 - 98.6]
PPV (%)	75.7 [66.8 - 82.8]	89.3 [78.5 - 95.0]	91.7 [78.2 - 97.1]
NPV (%)	82.3 [74.2 - 88.2]	65.2 [56.7 – 72.7]	54.1 [45.7 – 62.3]

## Discussion

The gold standard for diagnosis of BCa is the cystoscopy. However, the cystoscopy is an invasive procedure for patients. Urine cytology is the most widely used conventional method in predicting BCa. The sensitivity of urine cytology is poor for detecting low grade BCa although it is useful for detecting high grade BCa and carcinoma *in situ*. Therefore, identification of additional potent urinary biomarkers are sought to establish earlier detection of BCa.

Recently, Reg family proteins have been focused as candidates of serum biomarkers for detecting some cancers. [13-19] To our knowledge, there are no prior reports describing the expression of HIP/PAP in relation to the occurrence of BCa. In this study, we demonstrated HIP/PAP protein was significantly expressed in BCa specimens and BCa cell lines but not in normal bladder tissues (Figure1). Furthermore, urinary levels of HIP/PAP in BCa patients were significantly higher than those in the control group including healthy volunteers and patients with benign urological diseases (Figure3A). Interestingly, urinary levels of HIP/PAP also correlated positively with bladder tumor size (T stage), recurrence- and progression-risk classifications of non-muscle invasive BCa (Figures 3B and 4). Although two previous reports proposed that Reg-I could be a marker for BCa, [20,24] the ELISA used in this study showed virtually no cross-reactivates with Reg-Iα and -Iβ proteins (Figure2), indicating that the urinary signal detected in the ELISA was HIP/PAP and not Reg-I. In this study, we investigated six cases of non-interstitial severe cystitis patients in the group of benign urological disease, and two in the six cases were false positive. Similar to the cases of other urinary markers such as NMP-22 and BTA, the false positive for HIP/PAP may be possibly identified in patients with benign inflammatory conditions. In addition, to eliminate the potential impact of hematuria in this ELISA kit, we investigated two cases of patients with Nutcracker syndrome in the group of benign urological disease. Although the above two patients had gross hematuria, the concentration of HIP/PAP in the urine of both two cases was less than 8.5 pg/mL. We therefore assume that this ELISA kit is not affected by hematuria.

Approximately 80% of newly-diagnosed BCa patients have non-muscle invasive tumors. [25] Regardless of endoscopic complete resection of such tumors, about 50% and 15% of these cases have the possibility of recurrence and progression within 5 years, respectively. [26] The 5-year overall survival (OS) of patients with non-muscle invasive BCa ranges from 82% to 95%, whereas that of cases with muscle invasion is only 50%. [27-29] Therefore, both diagnosis of BCa at an early stage and strict surveillance after initial treatments are crucial for improving the outcomes of BCa patients. According to the EORTC risk criteria for non-muscle invasive BCa, patients treated with an initial transurethral tumor resection are divided into groups at low, intermediate, and high risk for recurrence and progression. The current study showed urinary HIP/PAP levels in the intermediate recurrence-risk group to be significantly higher than those in the low recurrence-risk group (Figure4A). Furthermore, urinary HIP/PAP levels correlated positively with the grade of progression-risk groups (low, intermediate, high) (Figure4B). On the basis of these results, pre-treatment urinary HIP/PAP levels may be applied as a prognostic factor for recurrence and progression of non-muscle invasive BCa. On the other hand, the reliability of predictive biomarkers is attributed to high PPV and NPV. However, routine urinary markers (e.g., NMP-22, BTA) are not sufficiently accurate to detect BCa. As shown in Figure5, the AUC of HIP/PAP and NMP-22 on ROC analysis were 0.863 (95% CI, 0.814-0.912) and 0.729 (95% CI, 0.655-0.803), respectively. This suggests that urinary HIP/PAP could be a better urinary marker for BCa than the urinary NMP-22. Furthermore, as shown in Table2, the statistical analysis to estimate the precisions of the three urinary markers revealed that HIP/PAP was superior to NMP-22 and BTA in terms of sensitivity (80.2% vs. 52.1% and 34.7%) and NPV (82.3% vs. 65.2% and 54.1%). This characteristic of urine HIP/PAP may be useful for reducing false negative cases of suspected BCa. In particular, it may contribute to strict follow-up for higher risk groups (e.g., intermediate and high) among non-muscle invasive BCa. However, false positive cases may undergo unnecessary endoscopic examination due to the low PPV of HIP/PAP. Based on the potential of current urinary markers, we propose that examinations combining a number of urinary biomarkers would provide a higher accuracy in predicting BCa.

## Conclusions

Urinary HIP/PAP is considered to be a potentially useful urinary marker for early detection of BCa or predicting recurrence and progression after an initial therapy for non-muscle invasive BCa. A large scale study will be needed to establish the usefulness of this biomarker.

## Abbreviations

HIP/PAP: Hepatocarcinoma-intestine-pancreas/pancreatitis-associated protein; BCa: Bladder cancer; PPV: Positive predictive value; NPV: Negative predictive value; BTA: Bladder tumor antigen; NMP22: Nuclear matrix protein 22; Reg: Regenerating gene; IC: Interstitial cystitis; PBS: Painful bladder syndrome; ELISA: Enzyme-linked immunosorbent assay; ROC: Receiver operating curve; AUC: Area under the curve; EORTC: European Organisation for Research and Treatment of Cancer; OS: Overall survival; BPH: Benign prostatic hyperplasia; LOH: Late-onset hypogonadism; NGB: Neurogenic bladder.

## Competing interests

The authors declare that they have no competing interests.

## Authors’ contributions

YN, HK, TN and HK designed this study. YN and TM collected human samples. YN, TT and HK analyzed the data. YN, HK, TT and HK prepared the manuscript. JI provided an antibody, and gave suggestions for this work. All authors approved the final version of the manuscript.

## Pre-publication history

The pre-publication history for this paper can be accessed here:

http://www.biomedcentral.com/1471-2490/12/24/prepub
